# Gene signature and prognostic value of ubiquitination-related genes in endometrial cancer

**DOI:** 10.1186/s12957-022-02875-w

**Published:** 2023-01-07

**Authors:** Ziwei Wang, Shuangshuang Cheng, Yan Liu, Rong Zhao, Jun Zhang, Xing Zhou, Wan Shu, Dilu Feng, Hongbo Wang

**Affiliations:** Department of Obstetrics and Gynecology, Union Hospital, Tongji Medical College, Huazhong University of Science and Technology, Wuhan, Hubei 430022 People’s Republic of China

**Keywords:** Endometrial cancer, Ubiquitination-related genes, Prognostic model, Survival, Differentially expressed genes, Tumor microenvironment

## Abstract

Protein ubiquitination is closely related to tumor occurrence and development. The specific role of ubiquitination in endometrial cancer remains largely unclear. Therefore, we constructed a novel endometrial cancer prognostic model based on ubiquitination-related genes. We extracted the expression matrices of ubiquitination-related genes from the Cancer Genome Atlas database, upon which we performed univariate Cox regression and least absolute shrinkage and selection operator (LASSO) regression analyses to obtain 22 ubiquitination-related genes for the construction of the prognostic model. Survival, regression, clinical correlation, and principal component analyses were performed to assess the performance of the model. Drug sensitivity analysis was performed based on these ubiquitination-related genes. Finally, a prognostic nomogram was constructed based on the prognostic model to quantify patient outcomes. Survival, regression, clinical correlation, and principal component analyses revealed that the performance of the prognostic model was satisfactory. Drug sensitivity analysis provided a potential direction for the treatment of endometrial cancer. The prognostic nomogram could be used to effectively estimate the survival rate of patients with endometrial cancer. In summary, we constructed a new endometrial cancer prognostic model and identified 5 differentially expressed, prognosis-associated, ubiquitination-related genes. These 5 genes are potential diagnostic and treatment targets for endometrial cancer.

**Supplementary Information:**

The online version contains supplementary material available at 10.1186/s12957-022-02875-w.

## Introduction

Endometrial cancer is one of the most common malignant tumors of the female reproductive system, with an increasing annual incidence rate [1]. According to statistics, endometrial cancer has the fourth highest incidence rate among malignant tumors in women in the USA, with approximately 66,570 new cases each year; it is also responsible for 12,940 deaths each year [2]. Studies have shown that the occurrence and development of endometrial cancer are significantly associated with metabolic syndrome, a history of oral estrogen or tamoxifen intake, and Lynch syndrome [3–9]. Early-stage endometrial cancer patients are often treated with surgery and have good prognosis; however, some patients develop lymph node or distant metastases, which leads to poor prognosis in these patients [10]. In summary, endometrial cancer seriously harms women’s health and creates a huge economic burden. Therefore, exploring the specific mechanisms underlying endometrial cancer development and progression is essential for its diagnosis and treatment.

Proteins are the main executors of life functions and play an important role in the regulation of life activities. Protein posttranslational modifications, including protein phosphorylation, acetylation, methylation, glycosylation, and ubiquitination, play a vital role in regulating life activities [11]. Ubiquitination is a dynamic and reversible modification of target proteins through the action of E1 ubiquitin-activating, E2 ubiquitin-conjugating, E3 ubiquitin-protein ligase, and deubiquitinating enzymes [12, 13]. Ubiquitination is involved in the occurrence and development of many diseases. For example, the E3 ubiquitin-protein ligase, MARCH8, redirects the viral M2 protein from the plasma membrane to the lysosome for its degradation through ubiquitination, thereby enhancing the body’s resistance against the influenza A virus [14]. DUSP26 promotes aortic valve calcification by inhibiting the DPP4 ubiquitination and degradation process which is mediated by the E3 ubiquitin-protein ligase, MDM2 [15]. The E3 ubiquitin-protein ligase, SPOP, inhibits AKT kinase activity and tumor progression by promoting the ubiquitination and degradation of PDK1 [16]. The E3 ubiquitin-protein ligase, FBXO16, inhibits ovarian cancer progression by promoting the ubiquitination and degradation of hnRNPL [17]. However, the mechanisms of ubiquitination involved in the occurrence and development of endometrial cancer remain unclear. Therefore, we constructed an endometrial cancer prognostic model based on ubiquitination-related genes to explore the impact of ubiquitination-related genes on endometrial cancer prognosis.

In this study, by mining transcriptomic endometrial cancer data from the TCGA database and the ubiquitination-related gene set from the integrated annotations for Ubiquitin and Ubiquitin-like Conjugation Database (iUUCD), we constructed a novel prognostic endometrial cancer model based on ubiquitination-related genes. From a holistic perspective, we explored the impact of ubiquitination-related genes on endometrial cancer prognosis and screened out differentially expressed, prognosis-associated, ubiquitination-related genes, which are potential diagnostic and treatment targets for endometrial cancer.

## Materials and methods

### Collection of datasets

We downloaded the fragments per kilobase million (FPKM) RNA-seq data of 575 endometrial cancer patients from the TCGA database (https://portal.gdc.cancer.gov/); this included 552 tumor tissue specimens and 23 normal tissue specimens [18]. GRCh38.p13 was downloaded from the Ensembl Human Genome Browser and used to convert gene Ensembl IDs to gene names (http://asia.ensembl.org/index.html) [19]. We downloaded the clinical data of endometrial cancer patients, including follow-up time, survival status, age, sex, pathological grade, and FIGO stage, from the UCSC Xena database (https://xenabrowser.net/) [20]. Ubiquitination-related genes were downloaded from the iUUCD database (http://iuucd.biocuckoo.org/); this included 27 E1 ubiquitin-activating, 109 E2 ubiquitin-conjugating, and 1153 E3 ubiquitin-ligase genes [21]. The gene expression and drug sensitivity files required for drug sensitivity analysis were downloaded from the CellMiner database (https://discover.nci.nih.gov/cellminer/home.do) [22].

### Construction of the prognostic model

Using the limma package of the R software, we obtained the expression matrices of all ubiquitination-related genes and merged them with the clinical data of endometrial cancer patients. Next, we performed univariate Cox regression analysis to identify prognosis-associated ubiquitination-related genes. The prognosis-associated ubiquitination-related genes were selected based on the criterion of the *P* value being less than 0.01. Using the glmnet and survival packages of the R software, we performed least absolute contraction and selection operator (LASSO) regression analysis on the prognosis-associated ubiquitination-related genes. Finally, 22 ubiquitination-related genes were selected for the construction of the novel prognostic model. We divided endometrial cancer patients into high-risk and low-risk groups based on the risk score which was calculated as, risk score =$\sum_{i=1}^n coef(i)\ast x(i)$, where coef(i) and x(i) represent the estimated regression coefficient and the expression value of the ubiquitination-related gene, respectively.

### Survival, ROC, and risk curves and principal component analysis

Using the survival and survminer packages of the R software, we drew survival curves for the endometrial cancer high-risk and low-risk groups. Using the survival, survminer, and timeROC packages of the R software, we drew a multi-index ROC curve to evaluate the accuracy of the constructed model. Using the pheatmap package of the R software, we drew the risk curves of the high-risk and low-risk groups. Using the Rtsne and ggplot2 packages of the R software, we performed principal component analysis and tSNE analysis for the high-risk and low-risk groups.

### Independent prognostic and clinical correlation analyses

We used the survival package of the R software to evaluate the effects of age, pathological grade, FIGO stage, and risk score on prognosis. Using the ggpubr package in the R software package, we evaluated the correlation between the ubiquitination-related genes and patient age, pathological grade, and FIGO stage.

### Tumor immune microenvironment and KEGG enrichment analyses

We used the GSVA, limma, and GSEABase packages of the R software to perform ssGSEA on the high-risk and low-risk groups and to score the immune cell and immune-related functions of endometrial cancer patients. Using the limma, ggpubr, and reshape2 packages of the R software, we evaluated the differences in immune cell and immune-related functions between the high-risk and low-risk groups. Using the limma and ggpubr packages of R software, we drew a boxplot of the risk scores between different endometrial cancer immunotypes. We used the limma and estimate packages of the R software to score immune and stromal cell function in the tumor microenvironment. Then, using the limma, ggplot2, ggpubr, and ggExtra packages of the R software, we drew scatterplots to describe the correlation between immuneScore and risk score and that between stromalScore and risk score. Finally, we downloaded the c2.cp.kegg.v7.4.symbols.gmt gene set from the GSEA website and performed KEGG enrichment analyses for the high-risk and low-risk groups; then, we used the plyr, ggplot2, grid, and gridExtra packages of the R software to visualize the top 10 enrichment results.

### Correlation analysis of immunotherapy-related genes

Using the limma, ggplot2, ggpubr, and ggExtra packages of the R package, we drew a boxplot of immunotherapy-related gene expression levels between the high-risk and low-risk groups and analyzed the correlation between risk score and immunotherapy-related gene expression levels. We drew survival curves for the ubiquitination-related genes using the survival package of the R package.

### Drug sensitivity analysis and construction of the prognostic nomogram

Using the limma package of the R software, we identified differentially expressed ubiquitination-related genes in endometrial cancer. These genes were selected based on the criteria of logFC being greater than 1 and the *P* value being less than 0.05. Using the Venn package of the R software, we intersected differentially expressed ubiquitination-related genes and the 22 ubiquitination-related genes and drew the Venn diagram. We used the pheatmap package of the R package to draw a heatmap of the intersecting genes. Using the survival package of the R package, we drew a forest map using the results of the univariate Cox regression analysis conducted on the intersected genes. Using the impute, limma, ggplot2, and ggpubr packages of the R package, we performed a drug sensitivity analysis for endometrial cancer. Finally, we used the rms, foreign, and survival packages of the R package to perform multivariate Cox regression analysis and drew prognostic nomograms for all the endometrial cancer samples; then, using the survival and timeROC packages of the R package, we drew a multi-index ROC curve.

### Data statistics

All statistical analyses were performed using the R version 3.6.1 software and strawberry-Perl-5.30.0.1. The values of *P* < 0.05 were considered statistically significant.

## Results

### Univariate Cox regression and least absolute contraction and selection operator (LASSO) regression analyses

**Fig. 1:**
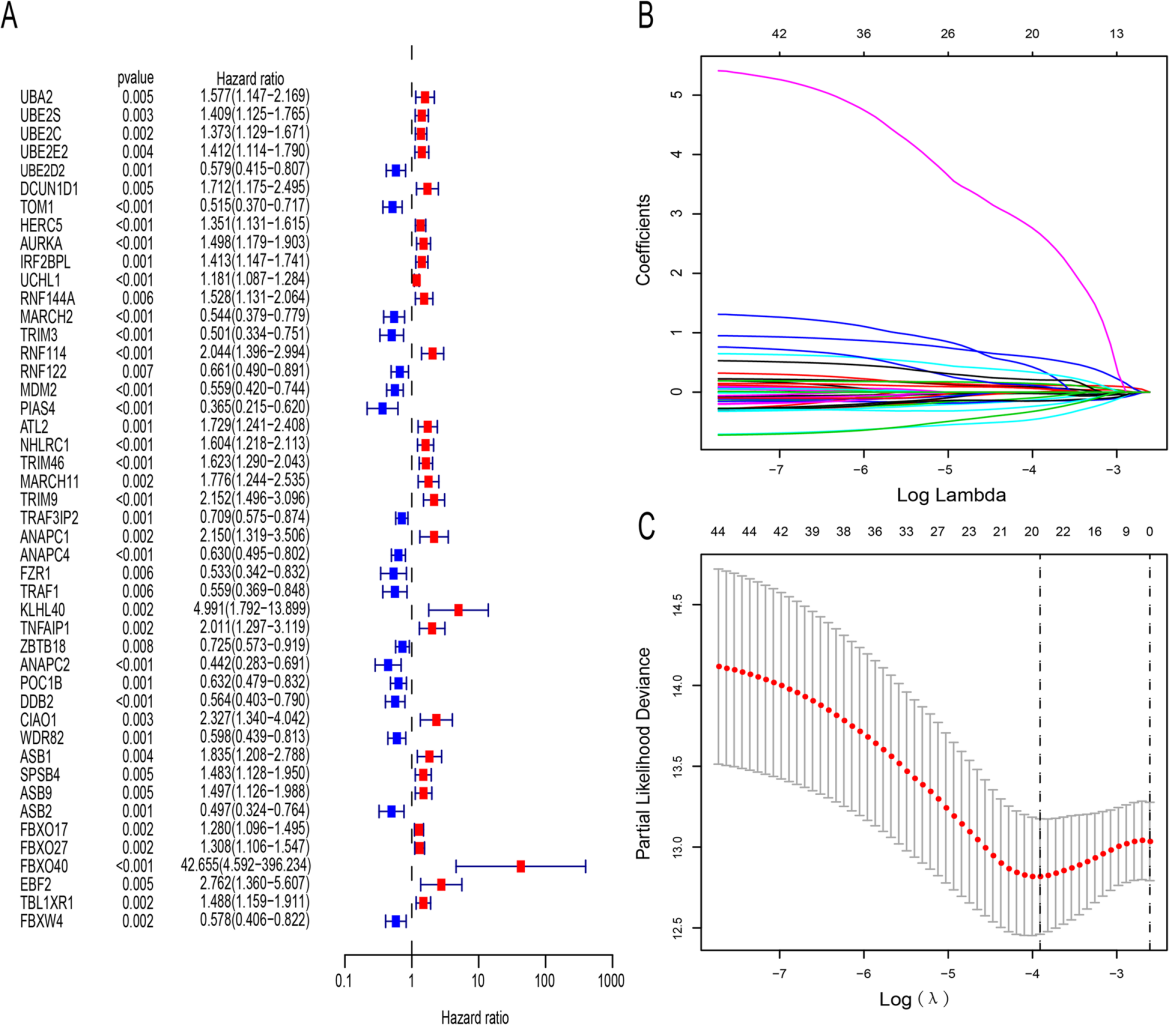
Univariate Cox regression and least absolute contraction and selection operator (LASSO) regression analyses. **A** Forest plot of univariate Cox regression analysis data. The *P* values, hazard ratios, and associated 95% confidence intervals for the ubiquitination-related genes are shown in the plot; red and blue represent the risk-associated ubiquitination-related genes (HR>1) and the protective ubiquitination-related genes (HR<1), respectively. **B** Distribution of LASSO coefficients between the 46 prognosis-associated ubiquitination-related genes. **C** Coefficient profile plot drawn against the log (lambda) sequence in the LASSO model. The optimal parameter (lambda) selected is represented as the first black dotted line

Ubiquitination plays a vital role in various tumors [23–25]. However, its role in endometrial cancer remains unclear. To explore the role of ubiquitination-related genes in endometrial cancer, these genes were analyzed by univariate Cox regression analysis, and 46 prognosis-associated ubiquitination-related genes were identified (Fig. 1A). As shown in Fig. 1A, the ubiquitination-related genes, UCHL1, FBXO17, FBXO27, HERC5, UBE2C, UBE2S , UBE2E2, IRF2BPL, SPSB4, TBL1XR1, ASB9, AURKA, RNF144A, UBA2, NHLRC1, TRIM46, DCUN1D1, ATL2, MARCH11, ASB1, TNFAIP1, RNF114, ANAPC1, TRIM9, CIAO1, EBF2, KLHL40, and FBXO40 were found to be risk genes, while PIAS4, ANAPC2, ASB2, TRIM3, TOM1, FZR1, MARCH2, MDM2, TRAF1, DDB2, FBXW4, UBE2D2, WDR82, ANAPC4, POC1B, RNF122, TRAF3IP2, and ZBTB18 were found to be protective genes. Then, we performed LASSO regression analysis on the 46 ubiquitination-related genes in order to construct an endometrial cancer prognostic model. As shown in Supplementary Table 1, 22 ubiquitination-related genes were used to construct the prognostic model. The calculation process of the regression coefficients is shown in Fig. 1B. The prognostic model performed best when all the 22 ubiquitination-related genes were included (Fig. 1C).

### Survival and principal component analyses

**Fig. 2:**
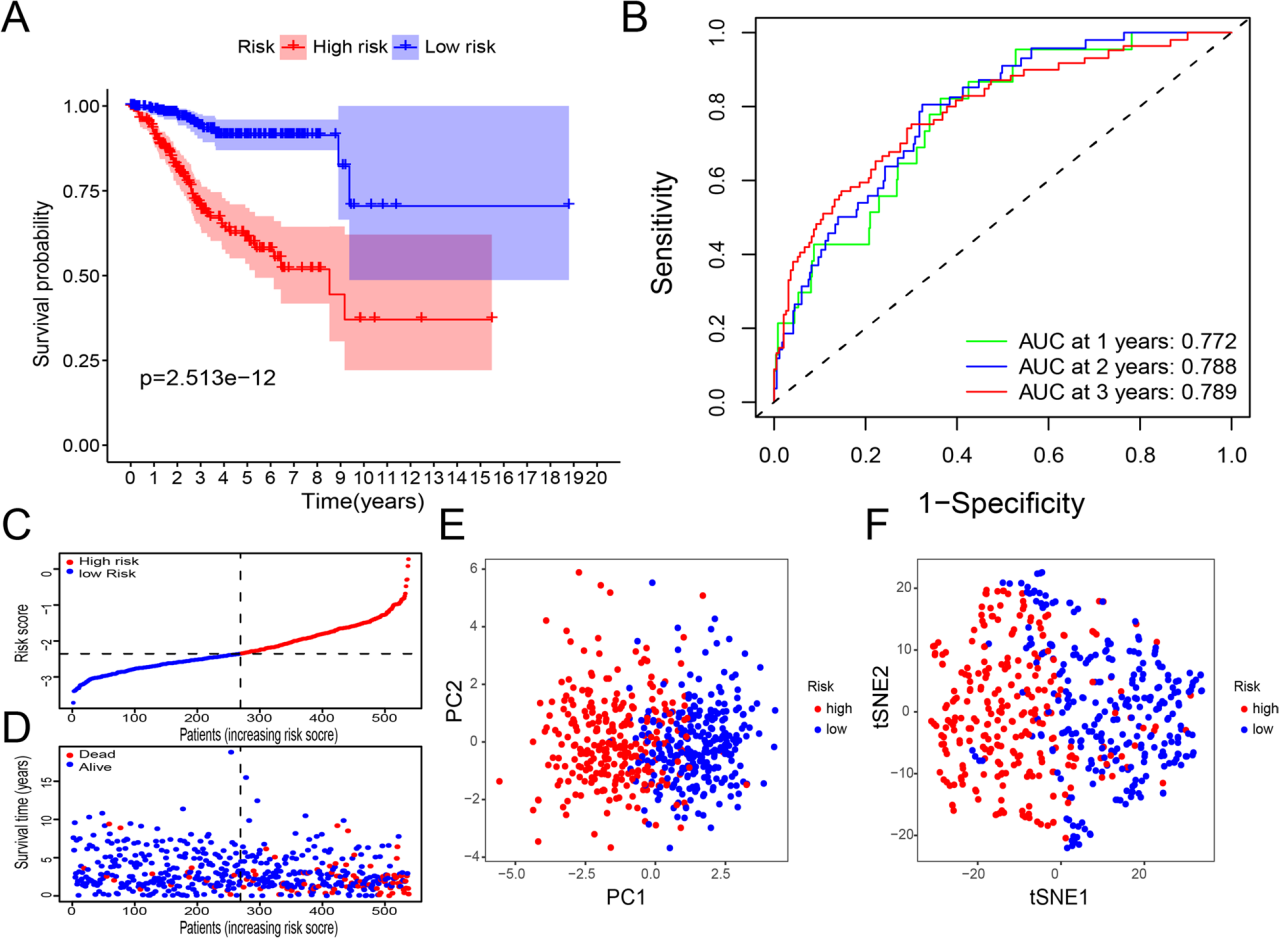
Survival and Principal Component Analyses results. **A** Survival curves for the high-risk and low-risk groups. Red and blue represent the high-risk and low-risk groups, respectively. **B** Multi-index ROC curve (AUC at 1 year=0.722, AUC at 2 years=0.788, and AUC at 3 years=0.789). **C** Risk curves for the high-risk and low-risk groups. Red and blue represent the high-risk and low-risk groups, respectively. **D** Scatter plot of different survival statuses. Red and blue represent dead and living ones, respectively. **E** Principal component analysis of the low-risk and high-risk groups. Red and blue represent the high-risk and low-risk groups, respectively. **F** t-SNE analysis of the high-risk and low-risk groups. Red and blue represent the high-risk and low-risk groups, respectively

Based on the 22 ubiquitination-related genes selected for the construction of the prognostic model, we divided all samples into the high-risk and low-risk groups based on the median risk score. Next, we drew survival curves for the high-risk and low-risk groups, and as shown in Fig. 2A, the survival rate of the high-risk group was significantly lower than that of the low-risk group. Then, we drew a multi-index ROC curve to evaluate the accuracy of the survival curve, and as shown in Fig. 2B, the AUC at 1, 2, and 3 years were 0.772, 0.788, and 0.789, respectively; thus, the survival curve was found to be moderately accurate. We drew risk curves for the high-risk and low-risk groups, and as shown in Fig. 2C, D, the risk score of the high-risk group was higher than that of the low-risk group, and the survival time and risk scores of dead patients were higher than those of living ones. Finally, we performed principal component and t-SNE analyses on the high-risk and low-risk groups, and as shown in Fig. 2E, F, the high-risk and low-risk groups were better separated.

### Evaluation of ubiquitination-related gene signatures as independent prognostic factors for endometrial cancer

**Fig. 3:**
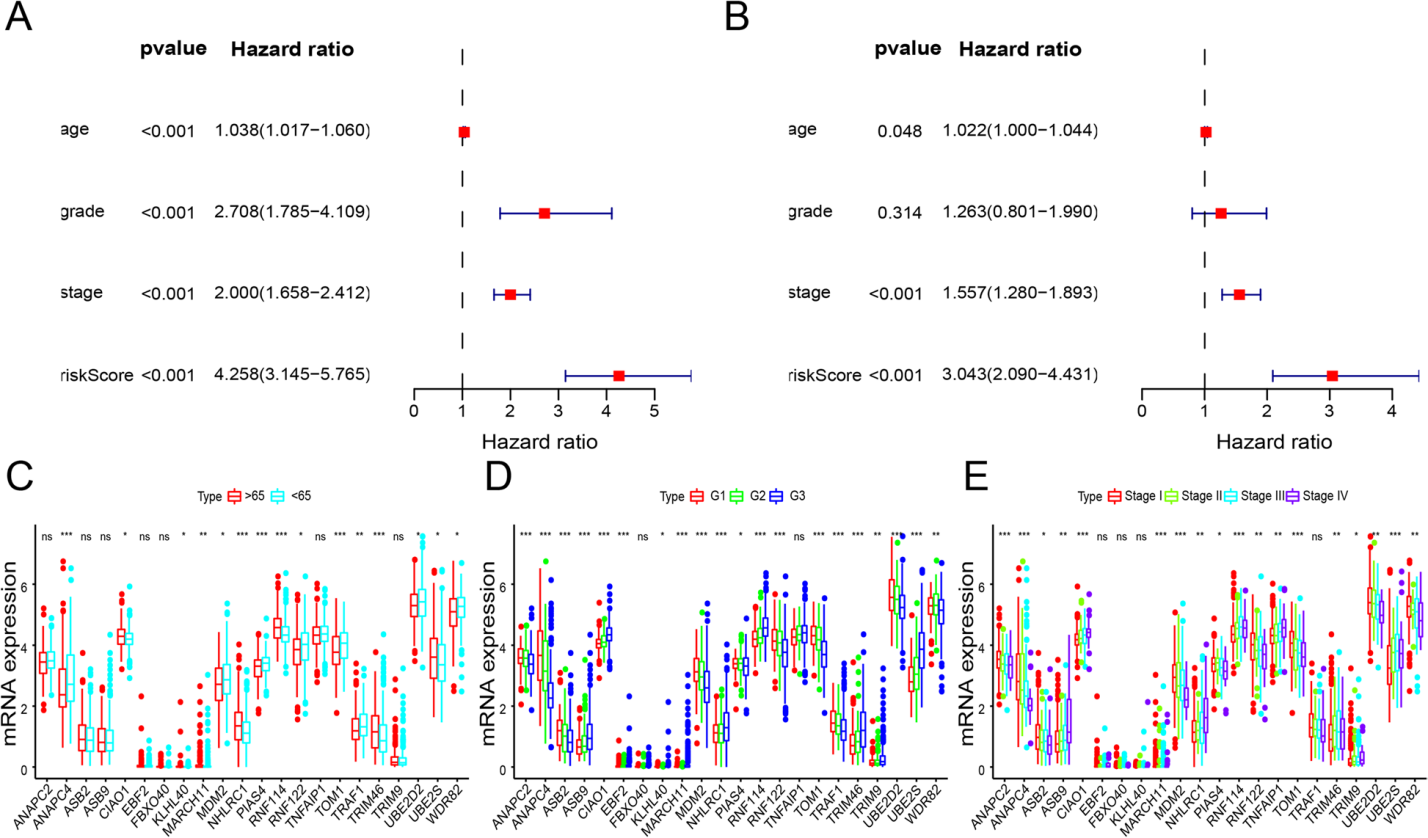
Evaluation of ubiquitination-related gene signatures as independent prognostic factors for endometrial cancer. **A** Univariate Cox regression analysis results. The *P* values, hazard ratios, and associated 95% confidence intervals are shown. Red and blue represent the risk-associated and protective factors, respectively. **B** Multivariate Cox regression analysis. The *P* values, hazard ratios, and associated 95% confidence intervals are shown. Red and blue represent the risk-associated and protective factors, respectively. **C** Ubiquitination-related gene expression levels in patients below and above 65 years of age. Red and blue represent the “below 65 years” and the “above 65 years” groups. **D** Ubiquitination-related gene expression levels in different pathological grades. **E** Ubiquitination-related gene expression levels in different FIGO stages. *p < 0.05, **p < 0.01, ***p < 0.001; ns: *p* > 0.05

Subsequently, we performed univariate and multivariate Cox regression analyses to evaluate the effects of clinicopathological characteristics and ubiquitination-related gene signatures on endometrial cancer prognosis and to determine whether ubiquitination-related gene signatures can be used as independent prognostic factors for endometrial cancer (Fig. 3A, B). As shown in Fig. 3A, through univariate Cox regression analysis, patient age, pathological grade, the International Federation of Gynecology and Obstetrics (FIGO) stage, and risk score were found to be prognostic risk factors. As shown in Fig. 3B, through multivariate Cox regression analysis, patient pathological grade, FIGO stage, and risk score were found to be prognostic risk factors for endometrial cancer. Then, we performed independent prognostic analyses on the 22 ubiquitination-related genes used to construct the prognostic model (Fig. 3C, E). The genes, ANAPC2, ASB2, ASB9, and TRIM9, were found to be associated with pathological grade and FIGO stage, while the genes, ANAPC4, CIAO1, MARCH11, MDM2, NHLRC1, PIAS4, RNF114, RNF122, TOM1, TRIM46, UBE2D2, UBE2S, and WDR82 were found to be associated with patient age, pathological grade, and FIGO stage. EBF2 and FBXO40 were associated with pathological grade, and KLHL40 and TRAF1 were associated with patient age and pathological grade, while TNFAIP1 was correlated with FIGO stage.

### Tumor microenvironment analysis and functional annotation

**Fig. 4:**
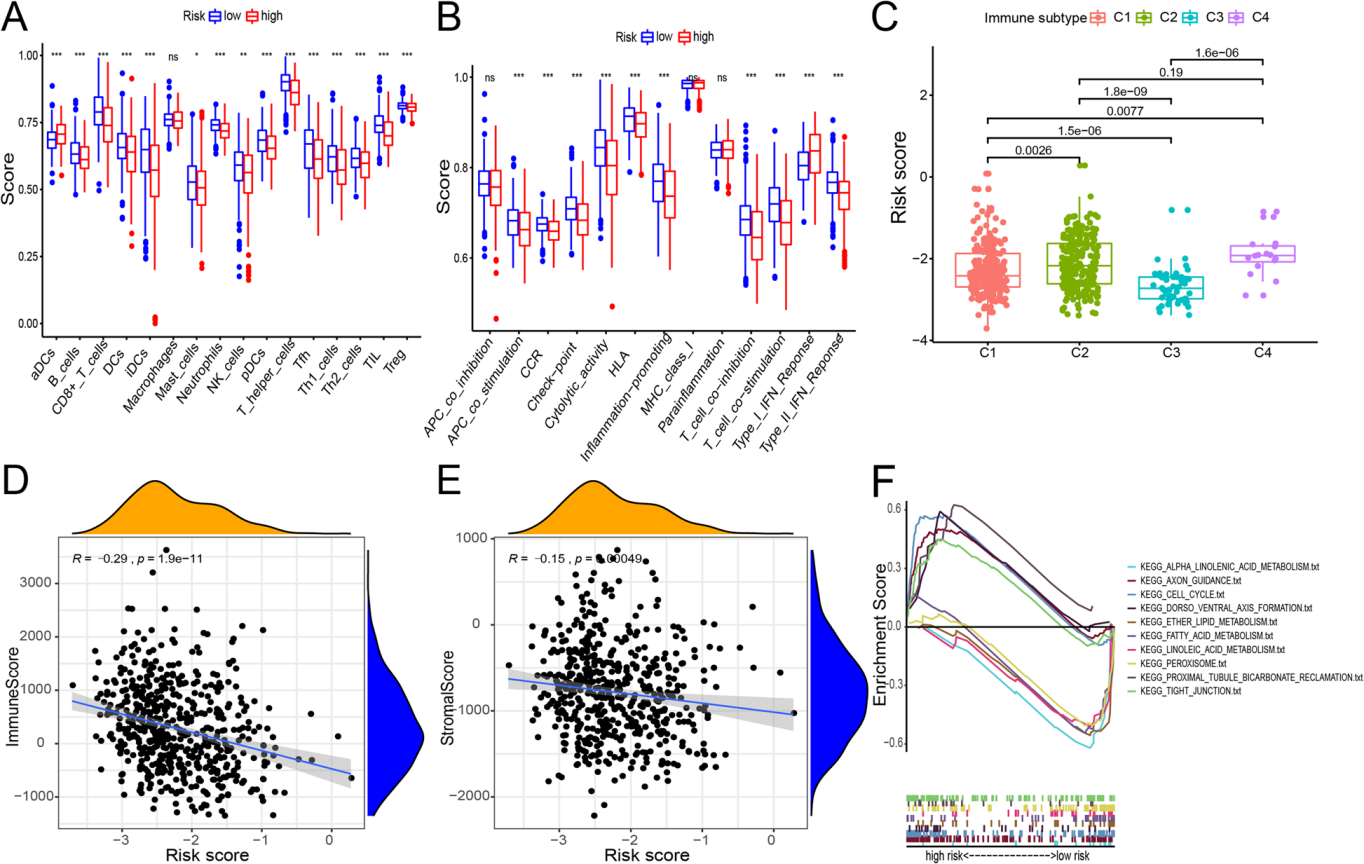
Tumor microenvironment analysis and functional annotation. **A**, **B** Immune cell scores and immune-related function score differences between the high-risk and low-risk groups. Red and blue represent risk-associated and protective factors, respectively. **C** Box plot of risk scores in the different endometrial cancer immunotypes. **D** Scatter plot of risk and immune scores. **E** Scatter plot of risk and stromal scores. **F** GSEA-based enrichment plot of the 22 ubiquitination-related genes in the high-risk and low-risk groups

Recent studies have shown that the tumor microenvironment plays a vital role in the occurrence and development of tumors [26, 27]. In this study, we analyzed changes in the tumor microenvironment using the prognostic model. Single-sample GSEA (ssGSEA) results showed that activated dendritic cells (aDCs) infiltration was higher in the tumor microenvironment of the high-risk group than in that of the low-risk group, while B cell, CD8+ T cell, dendritic cells (DC), immature dendritic cell (iDC), Mast cell, neutrophil, natural killer cell (NK cell), plasmacytoid dendritic cell (pDC), T helper cell, T follicular helper (Tfh), T helper 1 (Th1) cell, T helper 2 (Th2) cell, tumor-infiltrating lymphocytes (TIL), and regulatory T cell (Treg) infiltration in the tumor microenvironment of the high-risk group was lower than that in the tumor microenvironment of the low-risk group (Fig. 4A). Type I IFN response was higher in the high-risk group than in the low-risk group, while response to APC co-stimulation, chemokine receptor (CCR), check-point, human leukocyte antigen (HLA), inflammation-promotion, T cell co-inhibition, and type II IFN, and cytolytic activity, were higher in the low-risk group than in the high-risk group (Fig. 4B). Next, we analyzed the difference in risk scores between different endometrial cancer immunotypes. As shown in the Fig. 4C, the risk score of the wound healing group (immune C1) was significantly higher than that of the inflammatory group (immune C3), but lower than that of the IFN-gamma dominant (immune C2) and the lymphocyte depleted (immune C4) groups; the risk score of immune C2 was significantly higher than that of immune C3, while that of the inflammatory group was lower than that of the lymphocyte depleted group (Fig. 4C). Subsequently, we evaluated the correlation between risk score and immune score and that between the risk score and the stromal score. As shown on Fig. 4D, E, immune and stromal scores were negatively correlated with risk score. Finally, we performed a KEGG enrichment analysis on data from the high-risk and low-risk groups (Fig. 4F). The high-risk group was significantly more enriched in gene sets, KEGG_AXON_GUIDANCE, KEGG_TIGHT_JUNCTION, KEGG_CELL_CYCLE, KEGG_PROXIMAL_TUBULE_BICARBONATE_RECLAMATION, and KEGG_DORSO_VENTRAL_AXIS_FORMATION, than the low-risk group. The low-risk group was significantly more enriched in the gene sets, KEGG_ETHER_LIPID_METABOLISM, KEGG_ALPHA_LINOLENIC_ACID_METABOLISM, KEGG_FATTY_ACID_METABOLISM, KEGG_LINOLEIC_ACID_METABOLISM, and KEGG_PEROXISOME, than the high-risk group.

### Correlation analysis of immunotherapy-related genes

**Fig. 5:**
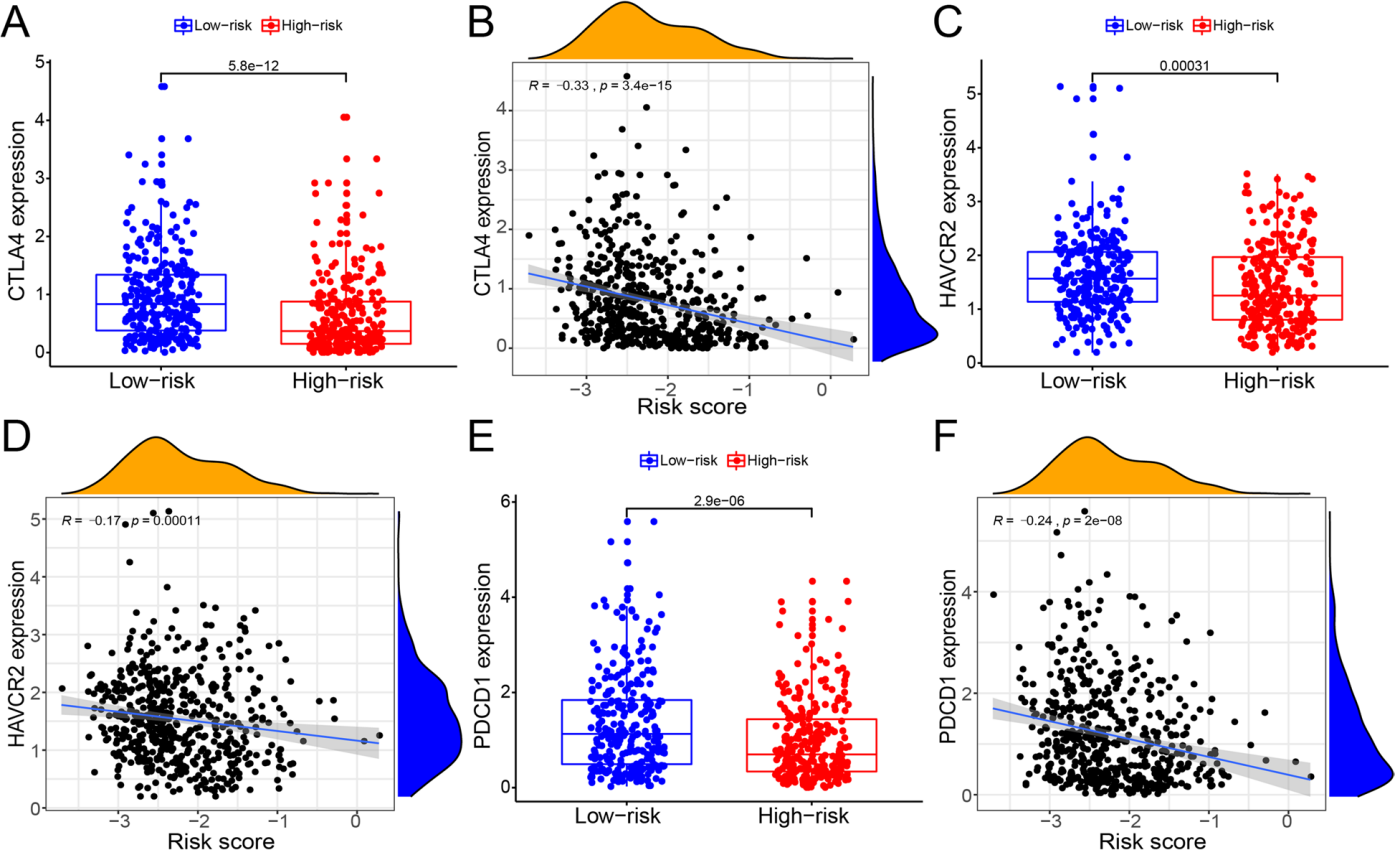
Correlation analysis of immunotherapy-related genes. **A** Boxplot of CTLA4 expression levels between the high-risk and low risk groups. Red and blue represent the high-risk and low-risk groups, respectively. **B** Scatter plot of CTLA4 expression levels and risk score. **C** Boxplot of HAVCR2 expression levels between the high-risk and low risk groups. Red and blue represent the high-risk and low-risk groups, respectively. **D** Scatter plot of HAVCR2 expression levels and risk score. **E** Boxplot of PDCD1 expression levels between the high-risk and low risk groups. Red and blue represent the high-risk and low-risk groups, respectively. **F** Scatter plot of PDCD1 expression levels and risk score

The advent of immune checkpoint inhibitors in recent times has significantly contributed to the treatment of malignant tumors. For example, the use of COX-2 inhibitors or EP2-4 PGE2 receptor antagonists in combination with immune checkpoint blockade (ICB) can increase effector T cell infiltration in the tumor microenvironment, thereby enhancing the anti-tumor effect [28]. In this study, we compared the expression levels of immunotherapy-related genes between different risk groups and evaluated the correlation between risk score and immunotherapy-related gene expression to establish a new basis for the immunotherapeutic treatment of endometrial cancer (Fig. 5A–F). We found that the expression levels of the immunotherapy-related genes, CTLA4, HAVCR2, and PDCD1, were significantly higher in the low-risk group than in the high-risk group and that the risk score was negatively correlated with the expression levels of the immunotherapy-related genes, CTLA4, HAVCR2, and PDCD1. Finally, we mapped survival curves for the 22 ubiquitination-related genes (Supplementary 1A-1F). The survival rate associated with the high expression genes, ASB2, TOM1, ANAPC4, and MDM2 was higher in the “high gene expression group” than in the “low gene expression group,” while that associated with the high expression genes, TRIM9 and TRIM46, was lower in the “high gene expression group” than in the “low gene expression group.”

*Construction of the prognostic nomogram*

**Fig. 6:**
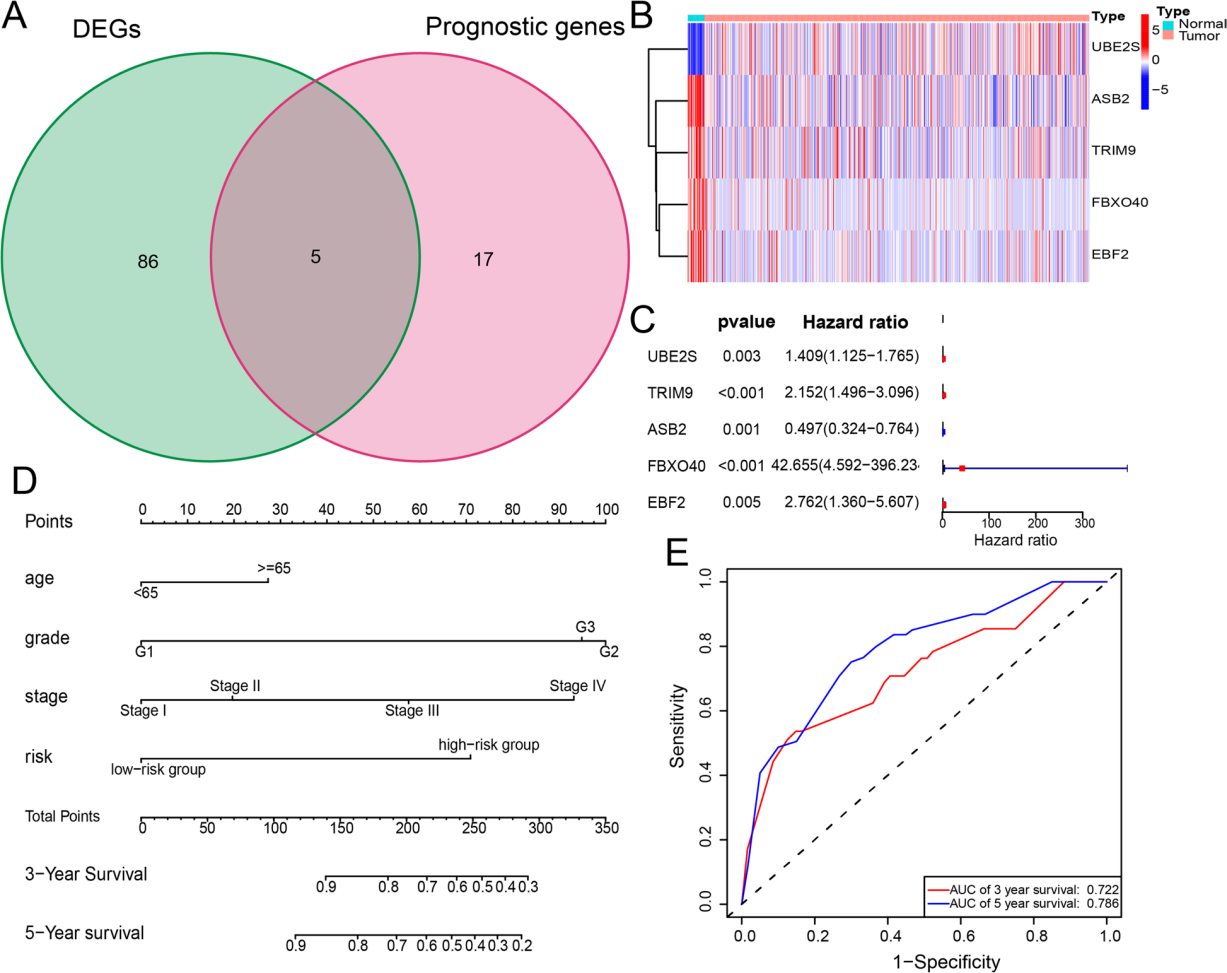
Construction of the prognostic nomogram. **A** Venn diagram showing the intersection between the differentially expressed ubiquitination-related genes and the 22 ubiquitination-related genes. **B** Heat map showing the differential expression of the intersecting genes. **C** Forest plot of the univariate Cox regression analysis data of the intersecting genes. The *P* values, hazard ratios, and associated 95% confidence intervals are shown in the figure. Red and blue represent risk and protective genes, respectively. **D** Prognostic nomogram. Each patient’s value is located on each variable axis, and a vertical upward line determines the number of points received for each variable value; the sum of these values is located on the “total points” axis, and a vertical downward line determines the likelihood of a 3- or 5-year survival. **E** Multi-index ROC curve for the nomogram. Red and blue represent the 3- and 5-year survival, respectively. The AUCs for the 3- and 5-year survival were 0.722 and 0.786, respectively

Subsequently, we screened out 91 endometrial cancer-associated differentially expressed ubiquitination-related genes and intersected them with the 22 ubiquitination-related genes, and 5 ubiquitination-related genes were obtained (Fig. 6A). Then, we drew a heatmap for the differential expression of these 5 ubiquitination-related genes, and as shown in Fig. 6B, the expression levels of UBE2S in tumor tissues were significantly higher than those in normal tissues, and the expression levels of ASB2, TRIM9, FBXO40, and EBF2 were higher in normal tissues than in tumor tissues. Next, we performed a univariate Cox regression analysis on the 5 ubiquitination-related genes, and as shown in Fig. 6C, UBE2S, TRIM9, FBXO40, and EBF2 were found to be risk genes, while ASB2 was found to be a protective gene. In summary, downregulating UBE2S expression or upregulating ASB2 expression in tumors is a potential treatment strategy for endometrial cancer. In this study, we conducted a drug sensitivity analysis on UBE2S and ASB2. As shown in Table 1, ASB2 was found to enhance endometrial cancer sensitivity to denileukin, diftitox, ontak, dabrafenib, vemurafenib, encorafenib, selumetinib, ARRY-162, vorinostat, cobimetinib (isomer 1), and imiquimod and to reduce its sensitivity to acetalax, umbralisib, bisacodyl (active ingredient of Viraplex), and floxuridine; UBE2S was found to enhance endometrial cancer sensitivity to floxuridine, cisplatin, gemcitabine, carboplatin, and bleomycin and to reduce its sensitivity to palbociclib, cobimetinib (isomer 1), and selumetinib (Table 2). These drugs may have a significant effect on the treatment of endometrial cancer. Finally, we treated the gene signatures of the 22 ubiquitination-related genes as independent prognostic factors, and multivariate Cox regression analysis was conducted for all the endometrial cancer samples (Table 2). We found that patient age, stage, grade, and risk score were risk factors for poor prognosis. Then, we drew a prognostic nomogram to calculate the survival rates of the patients based on the patient age, pathological grade, FIGO stage, and risk score (Fig. 6D). A multi-index ROC curve was plotted to evaluate the accuracy of the prognostic nomogram (Fig. 6E). We found that the AUCs of 3- and 5-year survival were 0.722 and 0.786, respectively. Thus, the prognostic nomogram was found to be moderately accurate.

**Table 1:** Drug sensitivity analysis

Gene	Drug	***Z*** score	***P*** value
ASB2	Denileukin Diftitox Ontak	0.423280461	0.000752632
ASB2	Dabrafenib	0.396122179	0.001730036
ASB2	Vemurafenib	0.374263231	0.003219909
ASB2	Encorafenib	0.327683507	0.0105937
ASB2	Acetalax	−0.326832776	0.010809518
ASB2	Selumetinib	0.324875662	0.011320505
ASB2	ARRY-162	0.322088064	0.012084319
ASB2	umbralisib	−0.315977652	0.013915612
ASB2	Vorinostat	0.297262357	0.021079614
ASB2	Cobimetinib (isomer 1)	0.291232828	0.023970188
ASB2	Bisacodyl (active ingredient of Viraplex)	−0.274871258	0.033546339
ASB2	Floxuridine	−0.256511757	0.047886716
ASB2	Imiquimod	0.256171913	0.04819329
UBE2S	Floxuridine	0.375855986	0.003081732
UBE2S	Cisplatin	0.351177337	0.005937641
UBE2S	Gemcitabine	0.303521756	0.018397248
UBE2S	Palbociclib	−0.300707931	0.019564728
UBE2S	Carboplatin	0.273631893	0.034386075
UBE2S	Bleomycin	0.271453043	0.03590475
UBE2S	Cobimetinib (isomer 1)	−0.259597709	0.045175275
UBE2S	Selumetinib	−0.254450134	0.049771279

**Table 2:** Multivariate Cox regression analysis for clinical characteristics and ubiquitination-related gene signatures

Variable	Coefficient	HR	Lower .95	Upper .95	***P*** value
Age≥65	0.4371	1.5482	1.0081	2.378	0.045842
Grade 2	1.5991	4.9487	1.1116	22.032	0.035837
Grade 3	1.5173	4.5599	1.0586	19.641	0.04171
Stage II	0.3142	1.3691	0.6455	2.904	0.412779
Stage III	0.9209	2.5114	1.5128	4.169	0.00037
Stage IV	1.4898	4.4364	2.3667	8.316	3.36E-06
High-risk group	1.1342	3.1087	1.7123	5.644	0.000193

Gene names, drug names, *Z* scores, and *P* values are shown in the table

Age ≥ 65 was compared to age<65; grades 2 and 3 were compared to grade 1; stages II, III, and IV were compared to stage I; the high-risk group was compared to the low-risk group. Regression coefficients, *P* values, hazard ratios, and 95% confidence intervals of the clinical characteristics are shown in the table

## Discussion

Protein post-translational modifications are widely involved in the regulation of various life activities and play an important role in the occurrence and development of many diseases [29, 30]. For example, in triple-negative breast cancer, KAT6A promotes interactions between SMAD3 and TRIM24 by mediating SMAD3 acetylation and inhibiting interactions between SMAD3 and TRIM33, thereby promoting tumor progression by regulating the immune microenvironment [31]. In liver cancer, TSP50 promotes tumor cell aerobic glycolysis, in vitro cell proliferation, and in vivo tumor formation by promoting PKM2 acetylation [31]. KDM4A promotes HIF1α expression by inhibiting histone methylation in the HIF1α promoter region, thereby upregulating DDIT4 expression and activating the mTOR signaling pathway to promote tumor proliferation, migration, and invasion [32]. SMYD3 promotes S1PR1 expression by promoting histone methylation in the S1PR1 promoter region, thereby promoting the progression of malignant tumors [33]. Protein ubiquitination, a post-translational protein modification, has recently become a research hotspot as it plays a vital role in the regulation of biological functions and the occurrence and development of diseases [34, 35]. For example, in non-small cell lung cancer, KLHL38 overexpression promotes PTEN ubiquitination and activates Akt signaling, thereby promoting cell proliferation, migration, and invasion [36]. TRIM26 mediates TAB1 polyubiquitination to enhance TAB1 activation and subsequently activates the NF-κB and MAPK signaling pathways to promote inflammatory immune responses [37]. The E3 ubiquitin-protein ligase, CUL3, inhibits autophagy by mediating the ubiquitination and degradation of BECN1, thereby promoting tumor occurrence and development [38].

In this study, by integrating the transcriptomic data of endometrial cancer patients from the TCGA database and the ubiquitination-related gene set data from the iUUCD database, we identified 22 ubiquitination-related genes and constructed a prognostic model. Then, we intersected the 22 ubiquitination-related genes and the differentially expressed ubiquitination-related genes, and obtained 5 ubiquitination-related genes, which were UBE2S, TRIM9, ASB2, FBXO40, and EBF2; UBE2S is an E2 ubiquitin-conjugating enzyme that has cancer-promoting effects in various tumors [39, 40]. For example, by ubiquitinating β-catenin, UBE2S promotes its expression and promotes the occurrence and development of rectal cancer [41]. In endometrial cancer, UBE2S promotes the proliferation and migration of endometrial cancer cells through SOX6/β-Catenin signaling [42]. In this study, UBE2S expression levels in endometrial cancer tissues were found to be higher than those in normal tissues, and UBE2S was found to be closely associated with endometrial cancer prognosis. Our results are consistent with previous studies. Therefore, UBE2S is a potential diagnostic and therapeutic target for endometrial cancer. TRIM9 encodes an E3 ubiquitin-protein ligase; studies have shown that TRIM9 can promote the occurrence and development of uterine fibroids through the NF-κB signaling pathway [43]. In this study, the expression levels of TRIM9 in tumor tissues were found to be lower than those in normal tissues, and it was found to be a risk factor for poor endometrial cancer prognosis. ASB2 is an E3 ubiquitin-protein ligase which can inhibit growth and chromatin condensation to inhibit the occurrence and development of leukemia [44]. In this study, ASB2 expression levels in endometrial cancer tissues were found to be lower than those in normal tissues, and it was found to be a protective gene. Therefore, up-regulation of ASB2 expression is a potential treatment strategy for endometrial cancer. FBXO40 is an E3 ubiquitin-protein ligase, the role in tumors of which has not been clearly elucidated. In this study, FBXO40 expression levels in endometrial cancer tissues were lower than those in normal tissues, and it was found to be a risk factor for poor endometrial cancer prognosis. EBF2 is an E3 ubiquitin-protein ligase that can promote osteosarcoma occurrence and development [45]. In this study, we found EBF2 to be a risk gene for endometrial cancer, and its expression levels in tumor tissues were lower than those in normal tissues.

In summary, we constructed a novel endometrial cancer prognostic model based on ubiquitination-related genes and validated it through survival analysis, regression analysis, and independent prognostic analysis. Moreover, we evaluated the correlation between the prognostic model and the tumor microenvironment. However, due to incomplete clinical data in some databases, we were unable to conduct cross-database verification. We identified 5 ubiquitination-related genes as potential diagnostic, treatment, and prognostic targets for endometrial cancer. However, the role and mechanisms of these ubiquitination-related genes in the occurrence and development of endometrial cancer needs to be further investigated.

## Conclusion

Using mined transcriptomic endometrial cancer patient data from the TCGA database, we constructed a novel endometrial cancer prognosis model based on 22 ubiquitination-related genes. Based on this prognostic model, we performed survival, independent prognostic, and GSEA enrichment analyses and evaluated differences in the tumor microenvironment between the high-risk and low-risk groups. Of note, we identified 5 ubiquitination-related genes as potential diagnostic and treatment targets for endometrial cancer.

## Supplementary Information

**Additional file 1.** Supplementary data.

## Data Availability

We obtained RNA seq data from TCGA (http://cancergenome.nih.gov/) and clinical information from the UCSC Xena (https://xenabrowser.net). Ubiquitination-related genes were downloaded the iUUCD database (http://iuucd.biocuckoo.org/). Gene sets for functional annotation were downloaded from GSEA (http://software.broadinstitute.org/gsea/index.jsp).
