# Supplementary material for: Gene signature and prognostic value of ubiquitination-related genes in endometrial cancer
Source: World J Surg Oncol. 2023 Jan 7;21:3. doi: 10.1186/s12957-022-02875-w (PMC9824913; doi:10.1186/s12957-022-02875-w)
Supplement: Supplementary file 1 — Additional file 1. Supplementary data. [file 12957_2022_2875_MOESM1_ESM.docx]

Supplementary Table 1. least absolute contraction and selection operator (LASSO) regression analysis of 46 ubiquitination-related genes

| **Gene** | | **Type** | | **Coefficient** | |
| --- | --- | --- | --- | --- | --- |
| UBE2S | | E2 ubiquitin-conjugating enzyme | | 0.057128046 | |
| UBE2D2 | | E2 ubiquitin-conjugating enzyme | | -0.123522464 | |
| TOM1 | | E3 ubiquitin-ligase enzyme | | -0.00028721 | |
| RNF114 | | E3 ubiquitin-ligase enzyme | | 0.004261418 | |
| RNF122 | | E3 ubiquitin-ligase enzyme | | -0.015635686 | |
| MDM2 | | E3 ubiquitin-ligase enzyme | | -0.145810768 | |
| PIAS4 | | E3 ubiquitin-ligase enzyme | | -0.44586564 | |
| NHLRC1 | | E3 ubiquitin-ligase enzyme | | 0.081223974 | |
| TRIM46 | | E3 ubiquitin-ligase enzyme | | 0.118530411 | |
| MARCH11 | | E3 ubiquitin-ligase enzyme | | 0.141920528 | |
| TRIM9 | | E3 ubiquitin-ligase enzyme | | 0.57370206 | |
| ANAPC4 | | E3 ubiquitin-ligase enzyme | | -0.02508659 | |
| TRAF1 | | E3 ubiquitin-ligase enzyme | | -0.088265462 | |
| KLHL40 | | E3 ubiquitin-ligase enzyme | | 0.37635539 | |
| TNFAIP1 | | E3 ubiquitin-ligase enzyme | | 0.321062237 | |
| ANAPC2 | | E3 ubiquitin-ligase enzyme | | -0.149215673 | |
| CIAO1 | | E3 ubiquitin-ligase enzyme | | 0.102033193 | |
| WDR82 | | E3 ubiquitin-ligase enzyme | | -0.227762151 | |
| ASB9 | | E3 ubiquitin-ligase enzyme | | 0.05898868 | |
| ASB2 | | E3 ubiquitin-ligase enzyme | | -0.307121694 | |
| FBXO40 | | E3 ubiquitin-ligase enzyme | | 2.664685934 | |
| EBF2 | | E3 ubiquitin-ligase enzyme | | 0.205518689 | |

Gene names, functions, and coefficients are shown in the table.

Supplementary 1A-1F. Survival curves of the ubiquitination-related genes


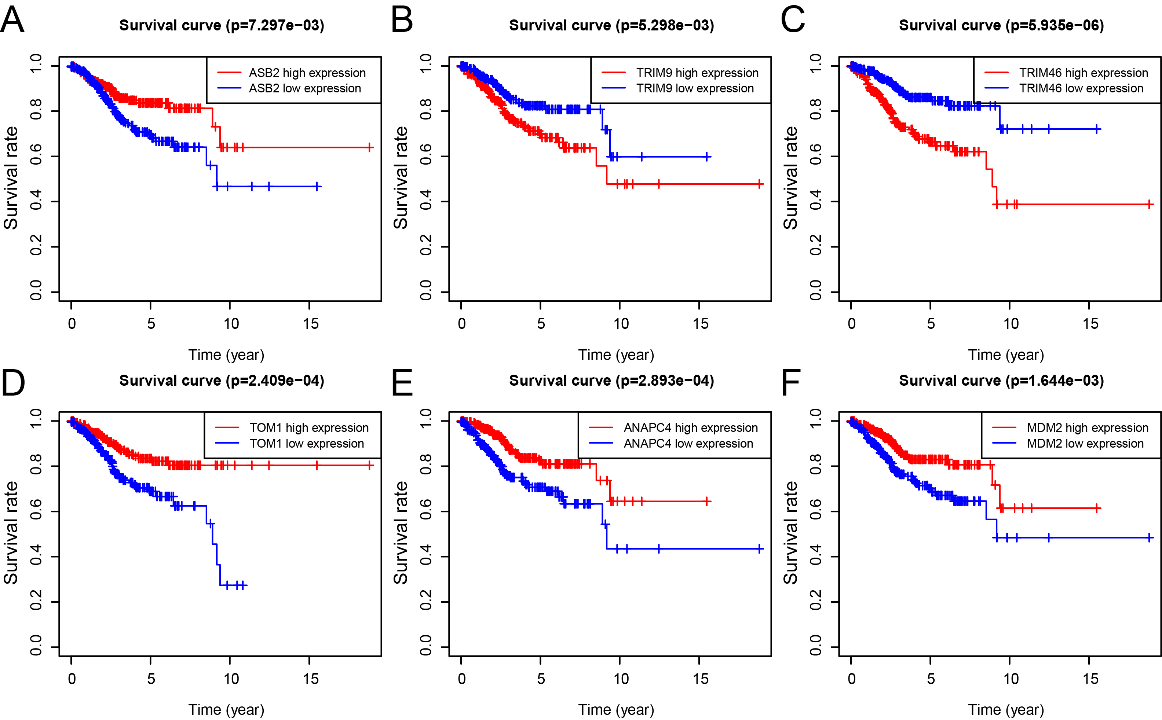


(A-F) Survival curves of the 22 ubiquitination-related genes. Blue and red represent the low expression and high expression groups, respectively.
